# P-985. Telementoring Program to Strengthen Antimicrobial Stewardship Programs (ASP) in Latin American Hospitals

**DOI:** 10.1093/ofid/ofaf695.1184

**Published:** 2026-01-11

**Authors:** Rodolfo E Quirós, Javier S Araujo, Alejandra Macchi, Eugenia Di Líbero, Miranda Teruel, María F Maldonado, Ana C Barbosa de Lima, Andrea Zurawski, Jorge Mera

**Affiliations:** CoNaCRA, CABA, Ciudad Autonoma de Buenos Aires, Argentina; Hospital Cuenca Alta Nestor Kirchner, CABA, Ciudad Autonoma de Buenos Aires, Argentina; Sanatorio Las Lomas, CABA, Ciudad Autonoma de Buenos Aires, Argentina; Hospital Evita de Lanús, CABA, Ciudad Autonoma de Buenos Aires, Argentina; Proanet, CABA, Ciudad Autonoma de Buenos Aires, Argentina; ECHO Institute – Health Sciences Center, University of New Mexico, Albuquerque, NewMexico; ECHO Institute – Health Sciences Center, University of New Mexico, Albuquerque, NewMexico; ECHO Institute – Health Sciences Center, University of New Mexico, Albuquerque, NewMexico; ECHO Institute – Health Sciences Center, University of New Mexico, Albuquerque, NewMexico

## Abstract

**Background:**

The ECHO Institute, in collaboration with PROAnet and Pfizer, developed a telementoring program to strengthen ASP in Latin American hospitals. This study assessed its impact on training outcomes and ASP development.

**Methods:**

A quasi-experimental, multicenter study was conducted across 80 hospitals in 10 countries. Hospitals performed self-assessments using the WHO qualitative and PROAnet quantitative (0-100 scale) tools, before and after intervention. A multivariate regression model identified institutional variables associated with ASP baseline levels. Between March and December 2024, 31 virtual sessions were held including 18 educational and 13 case-based sessions. Learning outcomes were evaluated through post-session surveys using Moore’s levels and 5-point Likert scales. Additionally, barriers and enablers to ASP implementation were also analyzed.

**Results:**

For-profit hospitals demonstrated higher scores than non-for-profits in ASP development (57.7 vs. 49.5; p=0.034), clinical guideline implementation (68.8 vs. 50.6; p=0.006), and antimicrobial prescription strategies (67.3 vs. 58.2; p=0.049). Multivariate analysis identified private funding (p=0.04) and continuous ASP implementation for >5 years (p< 0.01) as significant predictor of higher ASP development. Among the 73 hospitals with both baseline and post-study assessments, the overall ASP score showed a significantly increase (53.1 vs 64.0; mean difference 10.9; 95% CI 8.0 to 13.7; p< 0.0001), with notable improvements in Education, Training, and Safety Climate (39.0 vs 54.5; mean difference 15.4; 95% CI 11.3 to 19.5; p< 0.0001). Of the 2,166 participants, 34% were physicians, 24% pharmacists, 18% microbiologists, and 24% other specialists. Moore’s levels indicated high perceived relevance (87%), increased knowledge acquisition (97%), knowledge application (93%), ASP strategy implementation or modification (87%), and satisfaction (81%). A total of 22 enablers and 21 barriers to ASP implementation were identified.

**Conclusion:**

Institutional funding and ASP continuity were key determinants of success. The telementoring initiative significantly enhanced training and antimicrobial stewardship strategies, emphasizing the need for sustained institutional support and structured education.

**Disclosures:**

Ana C. Barbosa de Lima, Dr., Pfizer Inc.: Grant/Research Support Jorge Mera, Dr., Abbott Diagnostics: Grant/Research Support|AbbVie: Grant/Research Support|AbbVie: I am part of their speaker bureau and have received honoraria|Cepheid: Grant/Research Support|Gilead: Grant/Research Support

